# Genetic relatedness of multidrug resistant *Escherichia coli* isolated from humans, chickens and poultry environments

**DOI:** 10.1186/s13756-021-00930-x

**Published:** 2021-03-23

**Authors:** Mabel Kamweli Aworh, Jacob K. P. Kwaga, Rene S. Hendriksen, Emmanuel C. Okolocha, Siddhartha Thakur

**Affiliations:** 1 Department of Veterinary and Pest Control Services, Federal Ministry of Agriculture and Rural Development, Abuja, Nigeria; 2 Nigeria Field Epidemiology and Laboratory Training Programme, Abuja, Nigeria; 3 Department of Veterinary Public Health and Preventive Medicine, Ahmadu Bello University, Zaria, Nigeria; 4 Department of Population Health and Pathobiology, College of Veterinary Medicine, North Carolina State University, Raleigh, NC USA; 5 WHO, FAO, EU, Reference Laboratory for Antimicrobial Resistance, National Food Institute, Technical University of Denmark, Kgs. Lyngby, Denmark

**Keywords:** *Escherichia coli*, Antimicrobial resistance, Prevalence, Chicken, Multidrug resistance, Genetic relatedness, One health, Nigeria

## Abstract

**Background:**

Inappropriate use of antimicrobial agents in animal production has led to the development of antimicrobial resistance (AMR) in foodborne pathogens. Transmission of AMR foodborne pathogens from reservoirs, particularly chickens to the human population does occur. Recently, we reported that occupational exposure was a risk factor for multidrug-resistant (MDR) *Escherichia coli (E. coli)* among poultry-workers. Here we determined the prevalence and genetic relatedness among MDR *E. coli* isolated from poultry-workers, chickens, and poultry environments in Abuja, Nigeria. This study was conducted to address the gaps identified by the Nigerian AMR situation analysis.

**Methods:**

We conducted a cross-sectional study among poultry-workers, chickens, and poultry farm/live bird market (LBM) environments. The isolates were tested phenotypically for their antimicrobial susceptibility profiles, genotypically characterized using whole-genome sequencing (WGS) and in silico multilocus sequence types (MLST). We conducted a phylogenetic single nucleotide polymorphism (SNPs) analysis to determine relatedness and clonality among the isolates.

**Results:**

A total of 115 (26.8%) out of 429 samples were positive for *E. coli.* Of these, 110 isolates were viable for phenotypic and genotypic characterization. The selection comprised 47 (42.7%) isolates from poultry-workers, 36 (32.7%) from chickens, and 27 (24.5%) from poultry-farm or LBM environments. Overall, 101 (91.8%) of the isolates were MDR conferring resistance to at least three drug classes*.* High frequency of resistance was observed for tetracycline (n = 102; 92.7%), trimethoprim/sulfamethoxazole (n = 93; 84.5%), streptomycin (n = 87; 79.1%) and ampicillin (n = 88; 80%). Two plasmid-mediated colistin genes—*mcr-1.1* harboured on IncX4 plasmids were detected in environmental isolates. The most prevalent sequence types (ST) were ST-155 (n = 8), ST-48 (n = 8) and ST-10 (n = 6). Two isolates of human and environmental sources with a SNPs difference of 6161 originating from the same farm shared a novel ST. The isolates had similar AMR genes and plasmid replicons.

**Conclusion:**

MDR *E.coli* isolates were prevalent amongst poultry-workers, poultry, and the poultry farm/LBM environment. The emergence of MDR *E. coli* with novel ST in two isolates may be plasmid-mediated. Competent authorities should enforce AMR regulations to ensure prudent use of antimicrobials to limit the risk of transmission along the food chain.

**Supplementary Information:**

The online version contains supplementary material available at 10.1186/s13756-021-00930-x.

## Background

Antimicrobial resistance (AMR), is one of the biggest threats to food safety and considered a One-Health issue with the potential of spreading to other countries since resistant pathogens do not recognize boundaries [1, 2]. Recently, we have shown the transmission of AMR *E. coli* among chickens, humans, and the poultry environment [3, 4]. Globally, antimicrobial agents are used in food animal production to ensure good health and productivity of the animals [5–7]. Multiple studies have shown that inappropriate use of these antimicrobial agents in food animal production particularly poultry has led to the development of AMR [8–10].

Commensal *E. coli* are known to be part of the normal flora of the gastrointestinal tracts of man and animals without causing any harm to their host [11, 12]. Several *E. coli* strains have been used as indicator organisms in various studies on AMR [11, 13]. Although commensal *E. coli* are harmless to the host, the bacteria can acquire resistance genes and act as a reservoir for the spread of multidrug resistance (MDR) to and from food to humans [13]. The genetic structure of *E. coli* strains is usually influenced by several factors including the host and environment enabling the bacteria to acquire various AMR mechanisms [13, 14].

In September 2016, 193 member countries including Nigeria signed the United Nations General Assembly resolution to develop national action plans (NAP) on AMR [15]. In November 2016, Nigeria established its AMR coordinating body at the Nigeria Center for Disease Control (NCDC), and in January 2017, a One-Health AMR Technical working group was inaugurated to conduct AMR situation analysis and develop Nigeria’s NAP [16]. One of the data gaps identified from the AMR situation analysis was the paucity of AMR studies done in Nigeria across humans, food-producing animals, and the environment [16].

It has been documented that the continuous use of antimicrobial agents for therapeutic purposes against infections has led to the emergence of drug-resistant bacteria such as MDR *E. coli* [17]. MDR bacteria have made it difficult to treat certain infections effectively with modern or conventional antimicrobial agents [18]. AMR has resulted in treatment failure in human and animal populations, because of the emergence of MDR foodborne pathogens like *E. coli* arising from the abuse or misuse of antimicrobial agents [19]. This scenario further deteriorates in Nigeria because of the increasing number of farmers who practice self-prescription as well as self-administration of antimicrobials to their animals [5, 20]. Poultry farmers have easy access to antimicrobials that are available over-the-counter without prescription [3] and evidence shows that farmers administer the antimicrobials repeatedly against non-responsive infections [20, 21]. These actions by the farmers further promote the emergence and spread of antimicrobial-resistant foodborne pathogens with serious implications on public health [22]. Continuous administration of antimicrobial agents to chickens for prophylaxis, therapeutic, or growth promotion purposes increases the antibiotic selection pressure for resistance in the bacteria [23]. Our recent publication demonstrates that occupational exposure over ten years to chickens on poultry farms or live bird markets (LBMs) was a risk factor for MDR *E. coli* among poultry workers in Abuja [3].

We hypothesized that chickens harbouring MDR *E. coli* as well as contaminated poultry farm or LBM environment can become potential sources for transmission of resistance genes to poultry workers exposed to chickens and the environment on poultry farms or markets. To better understand the association between MDR *E. coli* isolates recovered from humans, chickens and poultry environment, we investigated the genetic relatedness of MDR *E. coli* isolates from poultry-workers, chickens, and selected poultry farms/LBM environments in Abuja, Nigeria.

## Methods

### Study overview

Our current study was part of a larger project conducted from December 2018 to February 2020 exploring MDR *E. coli* in humans, chickens, and the poultry farm/market environment. An aspect of this research exploring the risk factors for MDR *E. coli* among poultry workers has already been previously published [3]*.*

### Characterization of *E. coli* isolates

Of 429 samples collected in the course of the present study, 110 *E. coli* strains isolated from the stool of apparently healthy poultry workers, faecal samples obtained from chickens as well as from poultry litter and water obtained from farm and LBM environments were characterized. The sample collection procedures, isolation of *E. coli* from these samples, and antimicrobial susceptibility profiling of *E. coli* using the disk diffusion method have been described previously [3]. Briefly, suspected dark pink *E. coli* colonies on MacConkey agar were streaked on Eosin Methylene Blue agar and incubated at 37 °C for 24 h. Isolates were confirmed as *E. coli* using Microbact GNB 24E (Oxoid, UK).

### Genotypic Detection of *E. coli* isolates

#### Whole genome sequencing (WGS) of *E. coli* isolates

All isolates were subjected to WGS as previously described [4]. Briefly, libraries for each *E. coli* isolates were prepared for WGS using a Nextera XT kit. We processed 0.3 ng/µL of DNA from each isolate using a Nextera XT DNA Sample Prep Kit (Illumina Inc., San Diego, CA), pooled together, and sequenced on an Illumina Miseq platform using a 2 × 250 paired-end approach (Illumina Inc., San Diego, CA). Raw sequencing reads were de-multiplexed and converted to fastq files using CLC Genomics workbench version 9.4 (Qiagen bioinformatics, Valencia, CA). The DNA sequences for each isolate were transferred to the National Center for Biotechnology Information (NCBI) database after which each isolate was assigned an accession number.

### Bioinformatic analysis of WGS data

#### Antimicrobial susceptibility determinants of *E. coli* isolates

High-quality Illumina paired-end reads were assembled de novo into the draft genome sequence for each isolate using SPAdes assembler v.3.13.1 [24]. In silico detection and typing of resistance genes was done using ResFinder 3.2, a Center for Genomics Epidemiology (CGE) bioinformatics tool (database version 2020–02-11), to determine the acquired AMR genes as well as assess chromosomal point mutations [25]. For each isolate, we used between 95–100% identity to match individual genes to an annotated resistance gene. [25]. In silico determination of the existing plasmid replicon types of each *E. coli* isolate was done by submitting the assembled genomes to PlasmidFinder 2.1, a CGE bioinformatics tool (database version 2020–04-02). The selected threshold for minimum percentage identity was 95% while the minimum coverage of the contig was set at 60% [26]. The in silico plasmid MLST typing of replicons (*IncHI2 and IncF*) were determined by submitting the assembled genome to pMLST 2.0 (database version: 2020–04-20) bioinformatics tool on the CGE website [26].

#### Multilocus sequence typing (MLST) of MDR *E. coli* isolates

As previously described [4] in silico MLST-analyses of the *E. coli* isolates were determined using schemes demonstrated by Achtman which made use of allelic variation amongst seven housekeeping genes (*adk, fumC, gyrB, icd, mdh, purA, and recA)* to assign sequence types (STs) [27]. We used whole-genome sequence data to generate the *E. coli* MLST assignment for each isolate that perfectly matched the alleles in the MLST database. MLST Finder 2.0, a CGE bioinformatics tool was used to assign STs to the isolates with 100% match against known MLST alleles while those without perfect matches were identified as unknown [28]. Some isolates were assigned as a new type after matching with MLST alleles of unknown ST in the MLST database.

#### Determination of *E. coli* Phylogroups, SNPs calling and Phylogeny

Phylogroups of *E. coli* genomes were determined using an in silico Clermont typing method [29]. The Clermont Typer web interface is hosted by CATIBioMed (IAME UMR 1137) and accessible at http://clermontyping.iame-research.center/.

Phylogenetic trees were constructed to determine the phylogenetic relatedness of the *E. coli* isolates using the technique known as SNP calling described by Kaas et al. [30]. Briefly, the tool CSI Phylogeny, a CGE bioinformatics tool accessed online at https://cge.cbs.dtu.dk/services/CSIPhylogeny/ was used for SNP calling. The CSI phylogeny uses BWA to map raw reads to a reference sequence and uses Samtools for SNP calling. *E. coli* strain NCTC11129 was used as the reference strain for SNPs calling to identify variants present in the chromosome of each isolate. The selected thresholds used were: cut-offs for depth = 10x; SNP quality = 30; mapping quality = 25 and Z score = 1.96. The phylogenetic SNP-based maximum likelihood tree were annotated and visualized using the programs Figtree (http://tree.bio.ed.ac.uk/software/figtree/) and interactive Tree of Life tool—iTOL (http://itol.embl.de/itol.cgi). Pairwise SNP differences between genomes were computed to determine if isolates of different origins were related.

### Data analyses

Antimicrobial susceptibility data were analyzed using Epi Info 7 software by computing frequencies and proportions. The 108 assembled *E. coli* genomes of the present study have been deposited by the Thakur Molecular Epidemiology Laboratory, NC State University (GenomeTrakr Project) in the NCBI database under the Bioproject ID number PRJNA293225. The remaining two isolates have accession obtained from the DNA Data Bank of Japan (DDBJ) as previously reported [4].

## Results

### Antimicrobial susceptibility profile of *E. coli* isolates

A total of 110 *E. coli* strains were isolated from 122 human stool samples obtained from poultry workers on farms and LBMs; 111 faecal samples obtained from chickens on farms and LBMs; and 196 poultry litter and water samples obtained from farm and LBM environments. Of the 110 *E. coli* strains 42.7% (n = 47) were recovered from humans; 37.7% (n = 36) from chickens and 24.5% (n = 27) from poultry environment. High resistance rates were observed for tetracycline, trimethoprim/ sulfamethoxazole, streptomycin, ampicillin, nalidixic acid and gentamicin. On the contrary resistance to colistin, imipenem, ceftazidime, amoxicillin/clavulanic acid, cefuroxime, cefotaxime and ceftriaxone were quite low although colistin resistance rate of 11.8% in commensal *E. coli* is quite worrisome (Table 1).

**Table 1:** Antimicrobial resistance profiles of *E. coli* isolates from humans, chickens and farm/market environments in Abuja—Nigeria, 2019

Drug class	Drug	Resistance break point µg/mL	Human n = 47 (%)	Chicken n = 36 (%)	Environment n = 27 (%)	Total n = 110 (%)
Tetracyclines	Tetracycline	≤ 11	39 (83.0)	35 (97.2)	27 (100.0)	101 (91.8)
Folate Pathway antagonists	Sulfamethoxazole/Trimethoprim	≤ 10	39 (83.0)	31 (86.1)	24 (88.9)	94 (85.5)
Penicillins	Ampicillin	≤ 13	36 (76.6)	31 (86.1)	20 (74.1)	87 (79.1)
Quinolones	Nalidixic acid	≤ 13	26 (55.3)	27 (75.0)	19 (70.4)	72 (65.5)
Aminoglycosides	Streptomycin	≤ 11	35 (74.5)	30 (83.3)	22 (81.5)	87 (79.1)
	Gentamicin	≤ 12	20 (42.5)	27 (75.0)	16 (59.3)	63 (57.3)
Phenicols	Chloramphenicol	≤ 12	15 (31.9)	17 (47.2)	7 (25.9)	39 (35.5)
1^st^ Generation Cephalosporins	Cephalothin	≤ 14	13 (27.7)	15 (41.7)	5 (18.5)	33 (30.0)
Nitrofurans	Nitrofurantoin	≤ 14	5 (10.6)	13 (36.1)	8 (29.6)	26 (23.6)
Carbapenems	Imipenem	≤ 19	3 (6.4)	6 (16.7)	3 (11.1)	12 (10.9)
B-lactam inhibitors	Amoxicillin-clavulanate	≤ 13	2 (4.3)	5 (13.9)	3 (11.1)	10 (9.1)
3rd and 4th Generation Cephalosporins	Ceftriaxone	≤ 19	3 (6.4)	1 (2.8)	1 (3.7)	5 (4.6)
	Cefuroxime	≤ 14	4 (8.5)	3 (8.3)	0 (0)	7 (6.4)
	Cefotaxime	≤ 22	4 (8.5)	1 (2.8)	1 (3.7)	6 (5.5)
	Ceftazidime	≤ 17	4 (8.5)	2 (5.6)	5 (18.5)	11 (10.0)
Polymyxin	Colistin	≤ 11	7 (14.9)	3 (8.3)	3 (11.1)	13 (11.8)
Resistance to 3 or more classes of antibiotics	MDR	n/a	39 (82.9)	35 (97.2)	27 (100)	101(91.8)

**Fig. 1:**
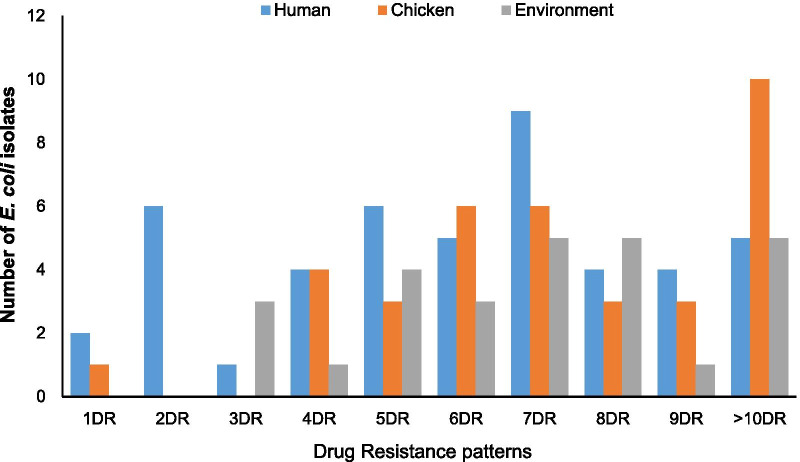
Antimicrobial resistance pattern of *E. coli* strains from humans, chickens, and poultry farm or market environment in Abuja-Nigeria, 2019. *DR means drug resistance; 1DR means the *E. coli* isolate was only resistant to one antimicrobial agent while > 10DR means the *E. coli* isolate was resistant to more than ten different antimicrobial drugs tested. The minimum number of antimicrobial drugs the human and chicken *E. coli* isolates were resistant to was one while the poultry environmental *E. coli* isolates were resistant to a minimum of three antimicrobial agents. Hence, all the poultry environmental *E. coli* isolates were multidrug-resistant

Analysis of resistance profiles of the 110 isolates showed that a single isolate (0.9%) from a poultry farmer was susceptible to all antimicrobial drugs tested; 4 (3.6%) were resistant to only one antimicrobial drug, 4 (3.6%) were resistant to two antimicrobial drugs and interestingly 101 (91.8%) were MDR (resistant to three or more classes of antimicrobial drugs). The number of antimicrobials against which each isolate showed resistance was between one and thirteen. Surprisingly, a single isolate from a poultry farm was resistant to 13 out of 16 antimicrobials tested. The AMR phenotypes with AMP, CEP, CHL, CT, GEN, NAL, S, SXT, and TET profile had the highest frequency of 13.6% (n = 15). Figure 1 summarizes the multiple AMR patterns exhibited by the isolates.

### Prevalence of MDR *E. coli* in humans, chickens and poultry farm/LBM environment

**Fig. 2:**
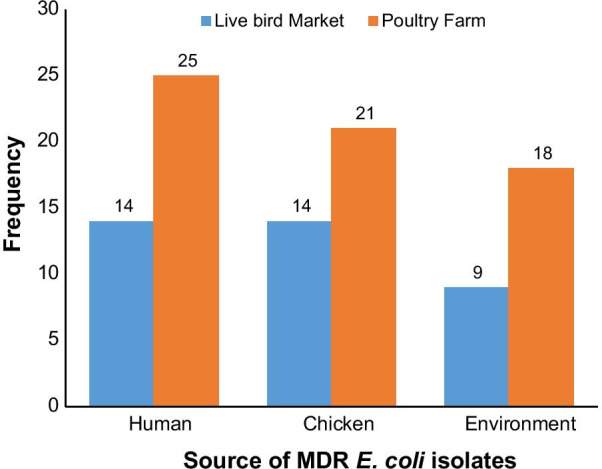
Distribution of MDR *E. coli* isolates based on isolation sources. The bars represent the number of MDR *E. coli* isolates from humans working on the poultry farm or selling chickens at the live bird market (LBM); the number of MDR *E. coli* isolates from chickens at the poultry farm or LBM and the number of MDR *E. coli* isolates from poultry farm or LBM environment

The overall prevalence of *E. coli* from all sources was 26.8% (n = 115), however, only 110 were further characterized due to viability as the remaining five isolates were mistakenly discarded. Of the 110 *E. coli* isolates, 91.8% (n = 101) were MDR *E. coli.* Of these MDR *E. coli* isolates 38.6% (n = 39), 34.7% (n = 35), and 26.7% (n = 27) were recovered from humans, chickens and poultry environment respectively (Fig. 2). Surprisingly, all the poultry environment isolates were MDR. Of the 101 MDR *E. coli* isolates 47.5% (n = 48) were MDR5 (resistant to more than 5 classes) and 38.6% (n = 39) were classified as XDR (resistant to 8 or more classes i.e. extensively drug-resistant isolates). Overall, 36.6% (n = 37) of the isolates originated from the LBMs while 63.4% (n = 64) originated from farms. Of the 39 XDR *E. coli* isolates 41% (n = 16), 33.3% (n = 13), and 25.6% (n = 10) were recovered from chickens, humans and the poultry environment respectively.

### In silico AMR gene analysis of MDR *E. coli* isolates in humans, chickens and poultry environment

This study identified 57 different resistance determinants from 101 MDR *E. coli* isolates. Genes encoding resistance to aminoglycosides accounted for the majority with about 14 different determinants (*aadA1*, *aadA2*, *aadA2b, aadA5*, *aadA16, armA, aac(3)-IIa*, *aac(3)-IId*, *aac(3)-Ib*, *aac(6)-Ib-cr*, *aph(3)-Ia*, *aph(3)-Ib*, *aph(6)-Id, ant(2)-Ia)* detected. A high prevalence (70.3%) of *aph(6)-Id,* which is a plasmid-encoded gene, was also observed. About two-thirds of the isolates (67.3%) exhibited *aph(3)-Ib* gene, a metabolic enzyme that confers aminoglycoside resistance. The *aac(3)-IId* gene responsible for conferring gentamicin resistance was observed in 27.7% of the MDR *E. coli* isolates. We also detected *aac(6)-Ib-cr* gene, responsible for the reduction in ciprofloxacin activity in two MDR *E. coli* isolates. Six different variants of β-lactam resistance genes were detected (*bla*TEM-1, *bla*OXA-1, *bla*OXA-10, *bla*OXA-129, *bla*CTX-M-15, *bla*CTX-M-65) out of which *bla*CTX-M type was classical of the ESBL producing *E. coli*. Ten different fluoroquinolone resistance determinants were observed, an important antimicrobial on the WHO list, (*qnrB1*, *qnrB19*, *qnrB52, qnrS1*, *qnrS2*, *qnrS3*, *qnrS7*, *qnrS11, qnrS13*, *aac(6)-Ib-cr)* and associated with mutations in the *gyr*A, *par*C, and *par*E genes. We detected other important resistance determinants such as trimethoprim resistance (*dfrA1*, *dfrA8, dfrA12*, *dfrA14*, *dfrA15, dfrA17, dfrA21, and dfrA27)*, macrolide resistance (*mdfA*, *mphA, mefB, ermB, ereA, mphE* and *msrE),* phenicol resistance (*cmlA1*, *catA1*, *catA2*, *catB3*, *floR)*, rifampicin resistance (ARR-2 and ARR-3), sulphonamide resistance (*sul1*, *sul2*, *sul3*), tetracycline resistance (*tet*A, *tet*B, *tet*M) and plasmid-mediated colistin resistance gene (PMCR)—*mcr*-1.1.

### Multi-locus sequence determination of MDR *E. coli* isolates

**Fig. 3:**
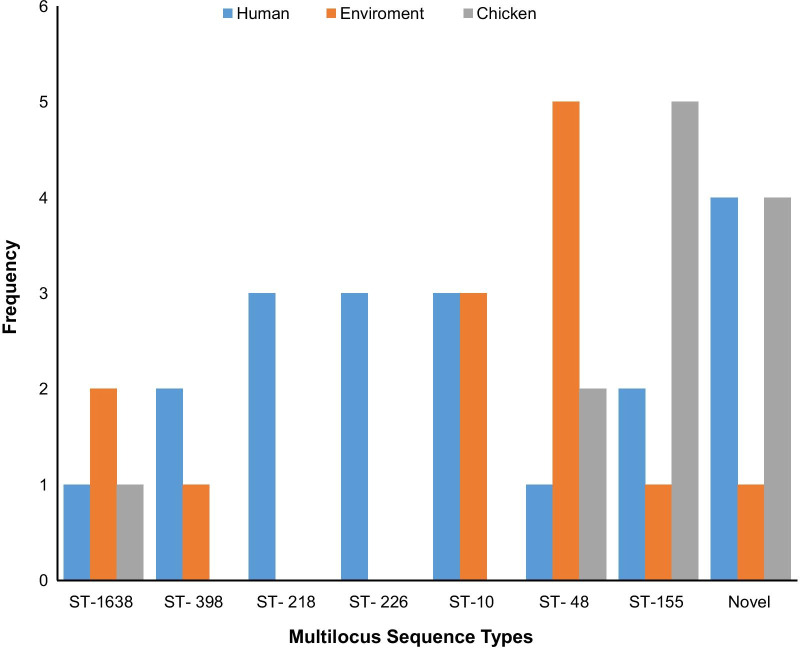
Multilocus Sequence Types for *E. coli* isolates from humans, chickens, and poultry farm/market environment, Abuja-Nigeria, 2019. Each bar represents the various *E. coli* sequence types for isolates obtained from humans, chickens, and poultry farm/LBM environments

The 101 MDR *E. coli* isolates belonged to 66 different sequence types (ST), out of which one (1) was non-conclusive and eight (8) were new types. In the in silico MLST analysis of *E. coli* isolates*,* the following were observed to appear more than once: ST155 (7.9%; n = 8), ST48 (7.9%; n = 8), ST10 (5.9%; n = 6), ST1638 (4%; n = 4), ST398 (3%; n = 3), ST216 (3%; n = 3), ST226 (3%; n = 3), ST101 (2%; n = 2), ST117 (2%; n = 2), ST165 (2%; n = 2), ST206 (2%; n = 2), ST4663 (2%; n = 2), ST1286 (2%; n = 2), and ST1196 (2%; n = 2). The most prevalent STs are shown in Fig. 3.

### Phylogroups of *E. coli* isolates from humans, chickens and poultry environment

**Fig. 4:**
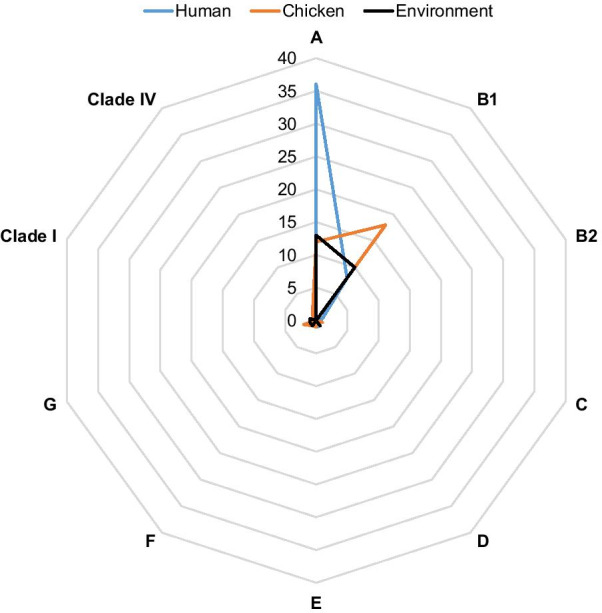
Phylogenetic classification of *E. coli* isolates from humans, chickens and poultry farm/market environments, Abuja-Nigeria, 2019. The peak of the blue triangle denotes the highest frequency of human *E. coli* isolates in phylogroup A. The orange triangle denotes the highest frequency of chicken *E. coli* isolates in phylogroup B1. The black triangle peaks in the same direction as the blue triangle indicating that the phylogroup A has the highest frequency for the *E. coli* isolates from the poultry farm/LBM environment

A majority of the isolates belonged to phylogroup A (n = 61, 55.5%) followed by phylogroup B1 (n = 36, 32.7%) while the rest belonged to phylogroup G (n = 3, 2.7%); D (n = 2, 1.8%); E (n = 2, 1.8%); F (n = 2, 1.8%); B2 (n = 1, 0.9%); C (n = 1, 0.9%); clade I (n = 1, 0.9%) and clade IV (n = 1, 0.9%). Isolates with phylogroup A originated from workers (n = 36) and poultry environment (n = 13) while isolates recovered from chickens mostly belonged to phylogroup B1 (Fig. 4). Of the 36 *E. coli* isolates, belonging to phylogroup B1, 22.2% (n = 8); 50% (n = 18) and 27.8% (n = 10) were recovered from humans, chickens and the poultry environment respectively.

All isolates assigned ST10 (n = 6), ST218 (n = 3), ST398 (n = 3) and ST1638 (n = 4) belonged to phylogroup A. However, all but one isolate assigned ST48 (7/8) and ST226 (2/3) also belonged to phylogroup A while a majority with ST155 (7/8) and novel ST (5/8) belonged to phylogroup B1.

### Plasmid replicon profiles of MDR *E. coli* isolates from humans, chickens and poultry environment

Forty (40) different plasmid replicon types were detected among 97 MDR *E. coli* isolates however, 4% (n = 4) did not harbour any plasmid replicons. The most prevalent plasmid replicons detected in descending order were p0111 (36.6%, n = 37); IncFIB(AP001918) (33.7%, n = 34); IncFII (18.8%, n = 19); ColpHAD28 (14.9%, n = 15); IncQ1 (13.9%, n = 14); IncFIB(K) (13.9%, n = 14); ColpVC (12.9%, n = 13); IncFIC(FII) (12.9%, n = 13); IncR (9.9%, n = 10); IncFII(pCoo) (9.9%, n = 10); IncY (9.9%, n = 10); IncX1 (8.9%, n = 9) and IncI1-I(gamma) (8.9%, n = 9). The plasmid replicons recovered from human isolates were more genetically diverse than those recovered from chickens and the poultry environment. Eighteen replicon types were common to isolates from all sources: p0111, IncFIB(AP001918), IncFII, ColpHAD28, IncQ1, IncFIB(K), ColpVC, IncFIC(FII), IncX1, IncFII(pCoo), IncI1-I (gamma), IncFII (29), IncFII(pHN7A8), IncFIA, Col156, IncHI2, IncHI2A and IncX4.

IncFIB(AP001918) was the most common among human isolates (n = 12) while p0111 was commonly detected in both chicken (n = 15) and poultry environment isolates (n = 14). Interestingly, IncFIB (pLF82), a phage plasmid was detected in one isolate recovered from the LBM environment. Eight different plasmids were observed to harbour AMR genes. The following AMR genes were carried on plasmid replicons: *mcr-1.1* + IncX4 (n = 2); *tetA* + IncX1 (n = 1); *qnrB19* + Col440I (n = 7); *sul2* + IncQ1 (n = 5); *aph(3)-Ib* + IncQ1 (n = 1); bla_TEM-1_ + IncFIC(FII) (n = 1); mdf(A) + IncFIB (n = 1); *qnrS13* + IncFII (n = 1) and aac(3)-IIa + IncHI1B (n = 1). The plasmid replicons harbouring the AMR genes was commonly detected in commensal *E. coli* isolates recovered from poultry workers, chickens and the poultry environment.

#### Determination of pMLST for IncHI2 and IncF plasmid replicons

In silico pMLST identification and typing of IncHI2 and IncF plasmid replicons, were based on the combination of the alleles identified for the genes. For the IncHI2 the assigned ST was ST4 for isolates (MA_251 and MA_252) originating from a poultry farmworker and poultry litter on the same farm. The pMLST analysis assigned the two IncF plasmids for isolates MA_251 and MA_252 with ST[F18:A-B1]. It is interesting to note that although the plasmid structures of the two isolates were so similar, there was no clonal relationship between them.

### Phylogenetic analysis of *E. coli* isolates from humans, chickens and poultry environments

**Fig. 5:**
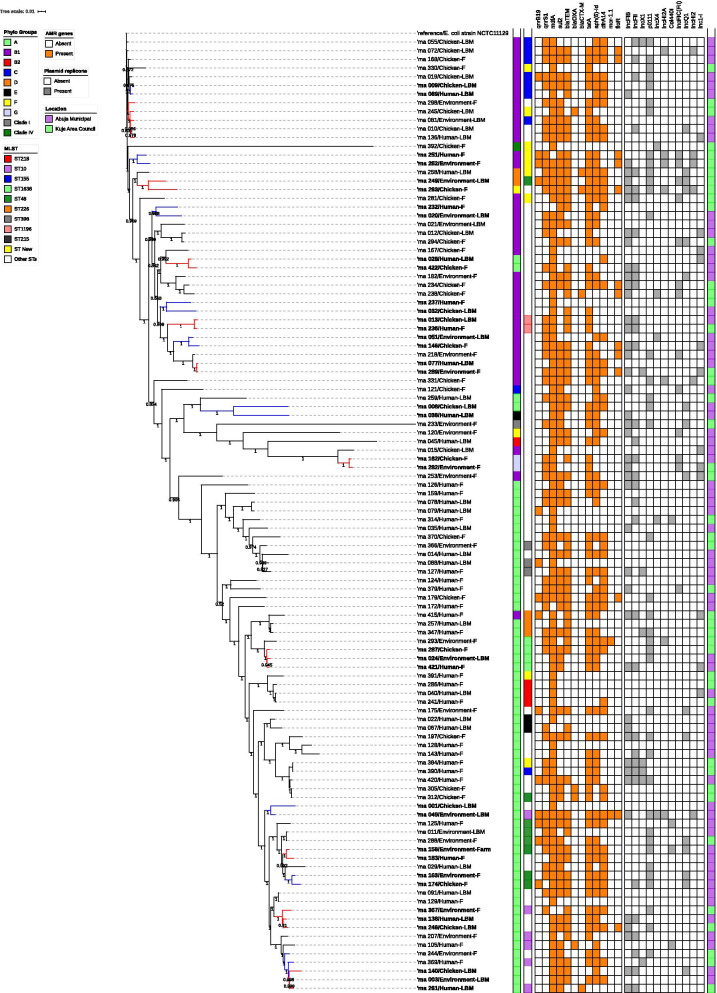
SNP-based phylogeny of MDR *E. coli* isolates from humans, chickens, and poultry environments in Abuja, 2019. SNP-based maximum likelihood phylogeny of *E. coli* isolates visualized in iterative Tree of life tool (iTol). The tree was rooted in a reference isolate *E. coli* strain NCTC11129. Clustering of isolates was found to be following the core genome and SNP-based phylogenies. The clustering of isolates belonging to the same phylogenetic group and sequence type was consistent. Shown for each isolate is the source/origin: farm (F) or live bird market (LBM) and phylogroup. AMR genes cluster for 110 *E. coli* strains are displayed on the phylogenetic tree. The presence (orange) and absence (white) of 12 AMR genes that were most prevalent are represented in the image while the presence (gray) and absence (white) of 10 prevalent plasmid replicons are also represented in the image

**Fig. 6:**
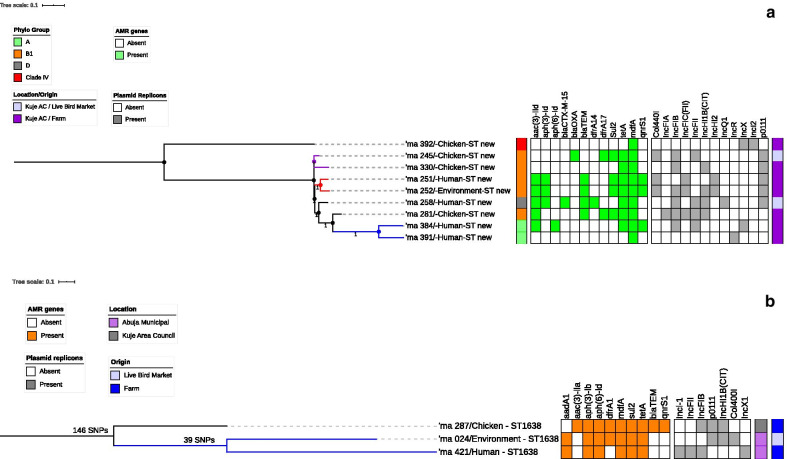
**a** Phylogenetic SNP-based maximum likelihood tree for *E. coli* isolates with Novel ST from humans, chickens and poultry farm or market environments. The phylogenetic SNP-based maximum likelihood tree was rooted in a reference isolate *E. coli* strain NCTC11129. For each isolate, the source and origin: farm (F) or live bird market (LBM) as well as the phylogenetic group is displayed. The phylogenetic tree has several clades with a common ancestor however the red clade has two isolates from the same farm belonging to the same phylogroup and sharing a novel ST. These two isolates from human and poultry farm environments although quite diverse had similar plasmid replicons harbouring AMR genes. **b** Phylogenetic maximum likelihood tree for *E. coli *isolates with ST 1638. The SNP-based maximum likelihood tree was rooted in a reference isolate *E. coli* strain NCTC11129. Two isolates of human and avian origin although not clonally related acquired similar AMR genes

All isolates assigned a phylogenetic group and ST were used to construct phylogenetic trees to determine if the isolates were genetically related or very diverse. Three phylogenetic trees were constructed: one for all the isolates (Fig. 5), one focusing on isolates with novel STs (Fig. 6a) and one with isolates of different origins assigned the same ST (Fig. 6b).

Overall, 110 isolates used to construct a maximum likelihood phylogenetic tree showed that the isolates were genetically diverse. The isolates were grouped based on similarities among them. Whole-genome (wg) SNPs-based phylogenetic analysis showed that some isolates sharing the same ST and phylogroups were not clonally related. The strains were in completely different clades in the SNP tree, separated by strains belonging to other STs. Three isolates with ST-1638 recovered from human, chicken and poultry environment were clustered together on the same clade. Pairwise SNP differences between the genomes of the isolates showed that they were not clonally related (Fig. 6b).

Two isolates of human and environmental origin with SNPs difference of 6161 were not clonally related although the isolates shared a novel ST and belonged to phylogroup B1 (Fig. 6a). The two isolates originating from the same farm had similar AMR gene profile (*qnrB19, qnrS1, mdfA, mefB, sul 2, sul 3, blaTEM1, tetA, tetM, floR*); as well as plasmid replicons (p0111, IncFIC(FII), IncHI2A, IncHI2, Col(pHAD28).

## Discussion

To the best of our knowledge, this is the first study to investigate the prevalence of MDR *E. coli* in poultry workers, chickens, and the poultry farm/market environments in Nigeria.

The first objective of this study was to characterize *E. coli* from poultry workers, chickens, and poultry environments. The unhygienic LBM environment where these chickens are sold acts as a reservoir of antimicrobial-resistant bacteria and eventually poses a health risk to people working in such an environment. A similar study done in the Netherlands reported a lower prevalence of MDR *E. coli* in chickens (23%) and chicken farmers (22%) when compared with the present study where a prevalence of 34.7% and 38.6% was detected in chickens and poultry workers respectively [31]. Access to antimicrobials is better regulated in the Netherlands as well as the implementation of antimicrobial stewardship programs when compared to Nigeria and could explain the differences observed in both studies. A related study conducted in Bangladesh among poultry and poultry environment reported a much higher prevalence of *E. coli* (82%) from chicken faecal samples when compared with the findings from this study, with a much lower prevalence of 32.2% [32]. Two similar studies performed among chickens from poultry farms in northern Nigeria also reported a much higher *E. coli* prevalence of 67.7% [33] and 69.8% [34] from cloacal swabs obtained from chickens on the farm. A possible explanation for the difference between studies carried out in northern Nigeria and our study could be due to the sample types collected as our study isolated *E. coli* from freshly dropped chicken faecal samples as opposed to cloacal swabs. A study conducted in Pakistan, reported a slightly higher *E. coli* prevalence of 36% from the poultry farm environment when compared to 26.1% obtained from the poultry environment in the present study [35]. Our study findings are consistent with the reports of a related study carried out in Egypt where *E. coli* prevalence of 26.8% was obtained from the poultry environment [36]. The similarity observed between our study findings and that of the Egyptian study may be due to similarities in poultry farming practices.

Our study examined AMR in *E. coli* isolates from poultry farm workers and chicken sellers and compared them to resistance rates of *E. coli* isolates from chickens and poultry farm/market environment. The patterns of resistance were similar for human and chicken isolates. High resistance rates were observed in isolates recovered from humans, chickens, and poultry farm/market environments for tetracycline, trimethoprim/ sulfamethoxazole, ampicillin, and streptomycin. This is in agreement with the findings of a study conducted in southwest Nigeria, where high resistance rates of *E. coli* isolates to beta-lactams, tetracyclines, macrolides, and sulfonamides were reported [37]. This finding is not surprising as these antimicrobials are easily accessible and commonly used in poultry production in Nigeria for therapeutic purposes especially in the absence of antimicrobial stewardship programs [38].

Our study revealed a very high proportion (91.8%) of MDR *E. coli* isolates from all the sources. Interestingly, 83% of human, 97% of chicken, and 100% of poultry environment isolates were MDR *E. coli*. A possible explanation for this very high level of resistance observed could be because of the lack of prudent use of antimicrobials and the required regulation to support it resulting in over-the-counter availability often without prescription as reported in many studies [16, 38–40]. The potential transmission of the drug-resistant strains between different hosts could also be responsible for this observation because *E. coli* is a known zoonotic bacteria [13].

The most common beta-lactamase gene observed in this study was the *bla*_TEM-1_ gene, which confers ampicillin resistance in *E. coli* isolates and is in agreement with a previous study that reported ampicillin-resistant *E. coli* isolates in food, humans, and healthy animals [41]. Our study however, did not detect any genes encoding carbapenem-hydrolyzing enzymes in any of the *isolates* although phenotypic characterization showed that 10.9% of the isolates were carbapenem-resistant. This may possibly be as a result of borderline interpretation of breakpoint settings between resistance and susceptibility. The present study identified one of the most important AMR genes [42], being the PMCR gene—*mcr 1.1* harboured on IncX4 plasmids in two isolates recovered from the poultry environment. Evidence shows that the IncX4 plasmids harbouring *mcr-1* genes have been detected in human and animal *E. coli* isolates however, our study recovered these plasmids from the poultry environment [43]. Another study conducted in China also detected PMCR genes—*mcr 1* in *E. coli* isolates sourced from the aquatic environment [44] however, the *mcr 3.1* gene was detected in a human *Salmonella* case in the US [45]. This further buttresses that *mcr* gene has spread across multiple pathogens.

Our study highlights that poultry workers, chickens, and the poultry environments share identical plasmid replicons and this is consistent with the literature [46, 47]. The IncF plasmids reported as one of the epidemic plasmids were observed in humans, chickens, and the poultry environments to harbour different AMR genes; *bla*_*TEM-1*_*, mdf(A)* and *qnrS13* in the present study and these are consistent with the literature [43]. The IncQ1 plasmids were detected in isolates with ST48 recovered from chickens and poultry farm environments harbouring the *sul2* genes that confer sulphonamide resistance and this is consistent with reports of other studies [43, 48]. The poor biosecurity measures, unhygienic practices in poultry farms and LBMs, and occupational exposure of over ten years are factors that predispose these humans to get infected with these drug-resistant bacterial strains [3].

To determine the genetic relatedness of the isolates, we analyzed by WGS, *E. coli* recovered from humans, chickens, and poultry environments. Our results revealed that these isolates showed very diverse genetic profiles. Common STs were assigned based on MLST including ST155, ST48, ST10, ST1638, and ST398 in isolates from humans, chickens, and poultry environments, although ST155 was mostly detected in isolates of poultry origin at the LBM. The most common ST detected among isolates recovered from the poultry farm environment was ST48. Previous studies have reported that *E. coli* with ST48 in phylogroup A has been detected in healthy volunteers, seafood, and water [49–51]. Our study detected ST48 in *E. coli* recovered from healthy people, chickens, and the poultry environment. *E. coli* strains with ST10 have previously been reported as being emerging and pathogenic as implicated in human infections although MDR strains with ST10 have also been detected in poultry and other animal sources [52]. Our study detected MDR *E. coli* strains with ST10 in healthy individuals, poultry manure, and water. A possible explanation could be that this is becoming an emerging public health issue arising due to possible mutations in the bacteria.

The majority of *E. coli* isolates in this present study belonged to phylogroup A (55.5%) and phylogroup B1 (32.7%). Most human and poultry environment isolates belonged to phylogroup A while majority of the chicken isolates belonged to phylogroup B1. Our study findings are in agreement with the results of a similar study conducted in Pakistan that reported that phylogroups B1 and A were the most prevalent detected among human and animal *E. coli* isolates [53]. Interestingly, a study carried out in south-west Nigeria reported that chicken *E. coli* isolates were evenly distributed into phylogroups A and B1 while phylogroup B1 was the most prevalent among human isolates [37]. Previous studies also showed that *E. coli* isolates belonging to phylogroup B2 are usually the most virulent, hence MDR [54–56]. However, our study observed that majority of the isolates, which belonged to either phylogroups A, and B1 were MDR. This is consistent with findings from a similar study conducted in south-west Nigeria which reported that isolates belonging to phylogroups B1 and A were MDR [37]. Our study findings are not surprising and consistent with the literature that most commensal *E. coli* belong to phylogenetic groups A and B1 [57, 58]. However, it is worrisome that these indicator bacteria have become MDR with a negative impact on public health since they could be transferred to more virulent strains or species thus causing disease.

The phylogenetic SNP tree rooted using NCTC11129 reference strain revealed that the isolates were genetically diverse among the identified STs. Two unrelated isolates of human and environmental origin belonging to phylogroup B1 and sharing a novel ST, had Col440I replicons harbouring the *qnrB19* genes that confer quinolone resistance and consistent with the literature [43]. In silico pMLST typing of the two isolates further confirmed that the isolates shared the same plasmids: IncHI2[ST-4] and IncF[ST-F18:A-:B1]. The two isolates although not clonally related, shared the same plasmids (Col440I) harbouring AMR genes (qnrB19) possibly due to horizontal gene transfer. Studies have shown that the IncF and IncHI2 plasmids mainly found in *E. coli* strains, are frequently detected in humans and animals serving as reservoirs for the spread of AMR genes and have been associated with MDR *E. coli* [43, 59]. This evidence supports our study results and explanation of a possibility of horizontal gene transfer of AMR genes harboured in the plasmids. Our study did not find evidence of the clonal spread of MDR *E. coli* at the human-animal-environment interface; however, our findings suggest that mobile genetic elements may have facilitated the horizontal transfer of MDR genes between the plasmids among commensal *E. coli* which could potentially mutate into real pathogens with serious public health implications [47].

## Conclusion

MDR *E.coli* isolates were found to be prevalent amongst poultry-workers, chickens, and poultry farm/market environments in Abuja, Nigeria. The highest resistance rates among MDR *E. coli* isolates were observed to tetracycline, sulphonamides, penicillins, aminoglycosides, and quinolones which are classes of antimicrobials commonly used in poultry production for treating avian diseases in Abuja. ST-155, ST-48, and ST-1638 were the only STs detected in humans, chickens, and poultry farm/LBM environments in our study. Our findings showed the emergence and spread of MDR *E. coli* with novel-ST from a poultry farm environment to a poultry farmer, which may have resulted from horizontal transfer of AMR genes harboured in plasmids. Consequent upon these, healthcare and poultry-workers should be educated on the fact that people in proximity with poultry are a high-risk group for faecal carriage of MDR *E. coli*. Competent authorities should enforce AMR regulation to ensure prudent use of antimicrobials to limit the risk of transmission along the food chain and to poultry workers. Farmers should be discouraged from indiscriminate use of antimicrobials in poultry production and encouraged to adopt preventive measures by observing biosecurity as well as good management practices on their farms.

## Supplementary Information

**Additional file1:** Supplementary Data.

## Data Availability

The datasets used and analyzed during the present study are available upon reasonable request from the corresponding author. All data generated or analyzed during this study are also included in this published article [and Additional file 1]. Raw sequence data have been submitted to NCBI (https://www.ncbi.nlm.nih.gov/) under accession no PRJNA293225.

## Abbreviations

AMR: Antimicrobial resistance
CGE: Center for Genomics Epidemiology
DDBJ: DNA Data Bank of Japan
DNA: Deoxyribonucleic acid
*E. coli*: *Escherichia coli*
iTOL: Interactive Tree of Life
LBM: Live bird market
MDR: Multidrug resistance
MLST: Multilocus sequence typing
NAP: National Action Plan
NCBI: National Center for Biotechnology Information
NCDC: Nigeria Center for Disease Control
pMLST: Plasmid multilocus sequence typing
PMCR: Plasmid-mediated colistin resistance
SNPs: Single nucleotide polymorphisms
SSR: Simple sequence repeat
ST: Sequence type
WGS: Whole-genome sequencing
Wg: Whole genome
